# Impact of Drug Safety Warnings and Cost-Sharing Policies on Osteoporosis Drug Utilization in Spain: A Major Reduction But With the Persistence of Over and Underuse. Data From the ESOSVAL Cohort From 2009 to 2015

**DOI:** 10.3389/fphar.2019.00768

**Published:** 2019-07-10

**Authors:** Isabel Hurtado-Navarro, Aníbal García-Sempere, Clara Rodríguez-Bernal, José Sanfélix-Genovés, Salvador Peiró, Gabriel Sanfélix-Gimeno

**Affiliations:** ^1^Fundación para el Fomento de la Investigación Sanitaria y Biomédica de la Comunidad Valenciana (FISABIO), Valencia, Spain; ^2^Red de Investigación en Servicios de Salud en Enfermedades Crónicas (REDISSEC), Valencia, Spain

**Keywords:** osteoporosis, bisphosphonates, drug labeling, cost-sharing, appropriateness, utilization

## Abstract

**Background:** Recent studies in several countries show a significant decrease in the consumption of osteoporosis drugs from a peak around 2009, mainly attributed to bisphosphonate safety warnings issued by regulatory agencies on jaw osteonecrosis, atypical fractures, and esophageal cancer, but no studies have assessed the impact of these warnings by risk of fracture strata.

**Aim:** The aim of this work is to assess changes in the utilization of osteoporosis drugs in the region of Valencia (Spain) after safety warnings from regulatory agencies and cost-sharing changes, according to patient socio-demographic and risk of fracture characteristics.

**Patients and Methods:** We constructed a monthly series of osteoporosis drug consumption for 2009–2015 from the ESOSVAL cohort (n = 11,035; women: 48%; mean age: 65 years old) and used interrupted time series and segmented linear regression models to assess changes in osteoporosis drug utilization while controlling for previous levels and trends after three natural intervention dates: the issue of the Spanish Agency for Drugs and Medical Products (AEMPS) Osteonecrosis Jaw Warning (Sept 2009), the AEMPS Atypical femur Fracture Warning (Apr 2011), and the modification of the cost-sharing scheme (Jul 2012).

**Results:** The AEMPS Osteonecrosis Jaw Warning was not associated with a decline in the consumption of osteoporosis drugs, while the warning on atypical fracture (a downward trend of 0.11% fewer people treated each month) and the increase in the cost-sharing scheme (immediate change level of -1.07% in the proportion of people treated) were associated with a strong decline in the proportion of patients treated, so that by the end of 2015 osteoporosis drug consumption was around half that of 2009. The relative decline was similar in people with both a high and low risk of fracture.

**Conclusion:** The AEMPS Atypical femur Fracture Warning of Apr 2010 was associated with a significant decrease in the number of people treated, reinforced by the increase in the pharmaceutical cost-sharing in 2012. Decreases in treatment affected patients both at a low and higher risk of fracture.

## Introduction

Osteoporosis is a common problem, particularly in the elderly population which is more prone to low-impact fragility fractures. Fragility fractures represent a major public health problem because of their contribution to disability, morbidity, mortality, and their cost for health care systems and society in general (Hernlund et al., 2013; Svedbom et al., 2013). Pharmacological secondary prevention after hip fracture—with bisphosphonates or alternative drugs—is recommended by virtually all clinical practice guidelines (CPGs) (Cosman et al., 2014; Allen et al., 2017; Compston et al., 2017) while pharmacological primary prevention is controversial (Järvinen et al., 2015) and CPGs are extraordinarily variable in their assessment of fracture risk factors, risk thresholds, drug risk assessment, and recommendations for pharmacological treatment in previously non-fractured patients (Bolland and Grey, 2010; Hurtado et al., 2014; Sanfélix-Genovés et al., 2014; Sanfélix-Gimeno et al., 2015; Wang et al., 2016). This uncertainty translates into a great variability in the use of osteoporosis drugs, which combines overuse (osteoporosis treatment in populations with a low risk of fracture, especially young adult women) and underuse (no treatment in men and women with a previous low-impact fracture or at a high risk of fracture) (Sanfélix-Gimeno et al., 2015; Fenton et al., 2016; Kyriakos et al., 2016).

While Spain is one of the European (and worldwide) countries with a lower incidence of osteoporotic fracture (Kanis et al., 2012; Hernlund et al., 2013), osteoporosis drug consumption experienced a very rapid growth during the 2000s (Salgueiro et al., 2013; Martín-Merino et al., 2017), Spain being one of the countries with the highest utilization rates at the end of that decade (Richards, 2010). For instance, the baseline data of the ESOSVAL cohort, recruited in 2009–2010, showed a prevalence of osteoporosis drug treatment of 28% in women aged 50 and over (Sanfélix-Genovés et al., 2013). Notwithstanding, recent studies in several countries show a significant decrease in the consumption of osteoporosis drugs from a peak in around 2009 (Peeters et al., 2014; Jha et al., 2015; van der Velde et al., 2017; Balkhi et al., 2018), including those for secondary prevention after hip fracture (Kim et al., 2016; Desai et al., 2018). This fall has been mainly attributed to safety warnings issued by regulatory agencies on jaw osteonecrosis, atypical fractures, and esophageal cancer (Ruggiero et al., 2004; Wysowski, 2009; Schilcher et al., 2011), and also to uncertainty about optimal bisphosphonate treatment duration and recommendations for discontinuation after 3 to 5 years of therapy, as the benefit-risk balance may become negative in the long term, particularly in patients with a low risk of osteoporotic fracture (Whitaker et al., 2012).

In this study, we hypothesize that safety warnings on oral bisphosphonates (the most widely prescribed osteoporosis drug class) issued by the Spanish Agency for Drugs and Medical Products (AEMPS) and the modification of the cost-sharing scheme (with both a 8–10% copayment for retired people who were previously exempt and increases in the copayment for most of the active working population) may have produced a reduction in the global prescription of osteoporosis drugs. Also, we hypothesize that, according to the fact that drug agencies maintained a positive risk-benefit balance in high-risk patients in their warnings, this reduction may occur mainly in people with a low risk of fracture [young people, without risk factors for secondary osteoporosis, without a previous fracture or with low-risk scores in the Fracture Risk Assessment Tool (FRAX^®^)], thus reducing overuse but keeping—or at least reducing to a lesser extent—appropriate prescription in high-risk patients. The aim of this work, using 2009–2015 data from the ESOSVAL prospective cohort, is to assess changes in the utilization of osteoporosis drugs in the Valencia Region (Spain) after the issue of safety warnings from regulatory agencies and cost-sharing changes, according to patient socio-demographic and risk of fracture characteristics.

## Materials and Methods

### Design

We use 2009–2015 data from the ESOSVAL prospective cohort to describe changes in osteoporosis drug consumption according to sociodemographic and clinical risk factors at baseline.

### Setting

The study was conducted in the VHS, an extensive network of public hospitals and primary healthcare centers which is part of the Spanish National Health System, funded and mostly provided by the Valencia Region Government, free at the point of care (except for some co-payments for out-of-hospital medication, increased in July 2012), and almost universal, covering about 97% of the region’s population (approximately 5 million inhabitants).

### Population

The ESOSVAL cohort, designed to develop a risk fracture assessment tool for the European Mediterranean population with a prevision of 10 years of follow-up, has been fully described elsewhere (Sanfélix-Genovés et al., 2010; Sanfélix-Genovés et al., 2013; Sanfélix-Gimeno et al., 2013; Hurtado et al., 2014) and was composed of about 11,000 people aged 50 years and over attending 272 primary healthcare centers in the Valencia Health System (VHS) for any health problem between November 2009 and September 2010. Participants were recruited by 600 general practitioners and primary care nurses collaborating for free in the ESOSVAL study and following predefined criteria attempting to obtain a similar number of men and women, and with an age distribution as close as possible to the distribution of the region’s population.

The baseline characteristics of the ESOSVAL cohort (n = 11,035; women: 48%; men: 52%; mean age: 65 years old) have been fully described elsewhere (Sanfélix-Genovés et al., 2013) and are summarized in **Table 1**. The exclusion criteria comprised temporary residents, individuals with cognitive impairment, people receiving their usual care through private insurance companies, people physically unable to attend their primary healthcare center, and people of Asian or African descent.

**Table 1:** Baseline characteristics of the ESOSVAL cohort at recruitment.

	Women		Men		All		
	50–64 n = 3,043	=65 n = 2,267	50-64 n = 2,983	=65 n = 2,742	50-64 n = 6,026	=65 n = 5,009	All N = 11,035
Educational level (% [95 CI])							
No studies	16.1 (14.7; 17.5)	50.6 (48.4; 52.7)	12.3 (11.1; 13.6)	42.4 (40.5; 44.4)	14.2 (13.3; 15.2)	46.1 (44.7; 47.6)	28.7 (27.8; 29.5)
Primary	50.5 (48.6; 52.4)	37.3 (35.3; 39.4)	45.1 (43.2; 47.0)	37.9 (36.0; 39.8)	47.8 (46.5; 49.2)	37.6 (36.2; 39.0)	43.2 (42.3; 44.2)
Second/university	33.4 (31.7; 35.2)	12.1 (10.8; 13.6)	42.6 (40.7; 44.5)	19.7 (18.2; 21.3)	37.9 (36.7; 39.2)	16.2 (15.2; 17.3)	28.1 (27.3; 29.0)
Personal history of previous osteoporotic fracture (% [95 CI])							
	6.6 (5.8; 7.5)	18.0 (16.5; 19.7)	3.3 (2.7; 4.0)	6.2 (5.3; 7.1)	5.0 (4.5; 5.6)	11.5 (10.7; 12.5)	8.0 (7.5; 8.5)
Falls (≥1 in the last year) (% [95 CI])							
	22.5 (21.0; 24.1)	30.0 (28.1; 32.0)	12.2 (11.0; 13.5)	18.6 (17.1; 20.1)	17.4 (16.5; 18.4)	23.7 (22.5; 24.9)	20.3 (19.5; 21.1)
Glucocorticoids use (prednisolone equivalent >5mg/day at least 3 months in the last year) (% [95 CI])							
	0.5 (0.3; 0.8)	1.7 (1.3; 2.3)	0.9 (0.6; 1.3)	1.5 (1.1; 2.0)	0.7 (0.1; 1.0)	1.6 (1.3; 2.0)	0.1 (0.0; 1.3)
Osteopenic diseases included in the FRAX tool excluded hypogonadism (% [95 CI])							
	9.5 (8.4; 10.6)	14.2 (12.7; 15.7)	10.4 (9.3; 11.6)	16.2 (14.8; 17.6)	9.9 (9.2; 10.7)	15.3 (14.3; 16.3)	12.3 (11.7; 13.0)
Hypogonadism (% [95 CI])							
	5.8 (4.9; 6.7)	5.8 (4.8; 6.9)	0.7 (0.4; 1.1)	1.5 (1. 1; 2.1)	3.3 (2.8; 3.8)	3.4 (2.9; 4.0)	3.3 (3.0; 3.7)
FRAX 10-years risk of hip fracture ≥3% (% [95 CI])							
	0.7 (0.4; 1.1)	41.6 (39.5; 43.7)	0.1 (0.0; 0.3)	19.3 (17.9; 20.9)	0.4 (0.2; 0.6)	29.4 (28.1; 30.7)	13.5 (12.9; 14.2)
Calcium and/or vitamin D supplements (% [95 CI])							
	20.6 (19.2; 22.1)	35.8 (33.8; 37.8)	2.4 (1.9; 3.0)	4.9 (4.1; 5.8)	11.6 (10.8; 12.5)	18.9 (17.8; 20.0)	14.9 (14.3; 15.6)
Antiosteoporotic treatment (any drug) (% [95 CI])							
	22.0 (20.5; 23.5)	36.3 (34.3; 38.3)	1.7 (1.3; 2.3)	3.1 (2.5; 3.8)	12.0 (11.2; 12.8)	18.1 (17.1; 19.2)	14.8 (14.1; 15.4)

### Data Sources and Study Development

The main source of data was the VHS ambulatory electronic medical record (EMR), which among other information includes demographic and clinical data and information on prescriptions and dispensations. In the context of the ESOSVAL project and in collaboration with the VHS, the ambulatory EMR was modified to include a specific osteoporotic risk sheet to facilitate the registration of fracture risk factors, patient monitoring, and decision-making about the need for complementary tests or pharmacological treatment. The EMR was modified for all VHS centers, but doctors and nurses participating in the ESOSVAL project were trained to standardize definitions and to fill in the EMR-specific osteoporotic risk sheet.

### Main Endpoint

Changes associated with the AEMPS safety warnings and cost-sharing changes in the monthly proportion of people filling any osteoporosis drug [bisphosphonates, calcitonin, denosumab, parathyroid hormone (PTH, 1–34 and 1–84), raloxifene, or strontium ranelate] between 1 Jan 2009 and 31 Dec 2015. Figures do not include zoledronic acid because inpatient based dispensation is not recorded in the ambulatory EMR, nor over-the-counter medication or treatments prescribed by private doctors not reimbursed by the VHS.

### Variables

The variables used in the present study include the patients’ sociodemographic and clinical baseline characteristics such as age, sex, educational level (no studies, primary studies, and secondary/university), and personal history of any previous osteoporotic fracture. Using the FRAX^®^ tool calibrated for Spain (https://www.sheffield.ac.uk/FRAX/) we estimated the 10-year risk of hip fracture for each patient (Kanis et al., 2008). Data in the FRAX^®^ web were introduced by the research team and calculations were based on gender, age, body mass index, personal history of previous fracture, family history of fracture, current smoking, glucocorticoid use, rheumatoid arthritis, other osteopenic diseases, alcohol intake, and bone mineral density (BMD) measurement, if available (women: 25.0%; men: 5.2%). In accordance with the FRAX^®^ recommendations, missing values were considered as normal. Although in Spain there are no official cutoff points for defining populations at a high or low risk of hip fracture, we tentatively use the criteria of the Scientific Advisory Council of Osteoporosis in Canada (Papaioannou et al., 2010) to classify the FRAX^®^ scores as low-risk (10-year risk of hip fracture <3%) or high-risk (10-year risk of hip fracture ≥3%).

### Statistical Analysis

First, we describe the baseline characteristics of the ESOSVAL cohort by gender and age groups at baseline (50–64, 65, and over) with the corresponding 95% confidence intervals calculated using the binomial approach. Second, we estimate the monthly proportion of patients treated with any osteoporosis drug (except zoledronic acid) according to sociodemographic and risk variables at baseline, and we calculate the risk ratio (RR) of being treated each month with respect January 2009 (the first month of the corresponding series). Considering the characteristics of the pharmaceutical package presentations authorized for osteoporosis treatment in Spain (almost all contain doses for 4 weeks or 1 month of treatment), we define “treated patients” as patients filling at least one package of any osteoporosis drug in the corresponding month, except for packages of ibandronic acid blister of 3-monthly tablets (we assume a 3-month coverage for that presentation) and denosumab (according to its recommended dosage, we assume a 6-month coverage for each package). Stockpiling was allowed for up to 1 month of treatment (e.g., for a patient filling two packages 1 month and none the next, both months were considered as covered by treatment).

Third, we used interrupted time series and segmented linear regression models to assess changes in osteoporosis drug utilization while controlling for previous levels and trends after three natural intervention dates: 1) the issue of the AEMPS Osteonecrosis Jaw Warning (ONJ warning, Sept 2009), 2) the issue of the AEMPS atypical femur Fracture Warning publication (AF warning, Apr 2011), and 3) the modification of the cost-sharing scheme on pharmaceuticals (Jul 2012). Trends are presented in natural scale (proportion of people treated) and in RR scale (ratio between the proportion of people treated each month and the proportion of people treated in January 2009) to compare the relative variations between strata in homogeneous terms. Model parameters and figures for the different segmented regressions are shown in the **Supplementary Files** (**Tables S2 to S11 and Figures S1 to S10**). Finally, in the supplementary files we analyze separately the annual consumption trends of the different osteoporosis drugs in terms of months of treatment dispensed each year, percentage of market share, and the annual ratio of dispensed treatments with respect to 2009 (**Supplementary Files Table S12 and Figures S11 and S12**).

In all analyses, people who died were excluded from the respective denominator in the month of death. Cases with missing data in one variable were eliminated from the analyses using that variable. All analyses were performed using the STATA 13.0 (StataCorp, College Station, TX) statistical software.

### Ethical Aspects

The ESOSVAL project is an observational study with no intervention components apart from the training of participating clinicians, and with no additional tests, visits, evaluations, or treatments provided apart from what the attending physician deemed appropriate. All patients included in the study signed the informed consent form granting the researchers access to the information contained in their medical record for the study purposes. The information relative to the patients was handled according to Spanish and European regulations on data protection and patients’ digital rights. The ESOSVAL project was approved by the Committee for Ethics and Clinical Trials of the Centre for Public Health Research and the Public Health General Directorate (decision March 27, 2009).

## Results

Mean age at recruitment was 64.3 (SD: 9.3) years for women and 65.6 (SD: 9.9) years for men, with 42.7% of the women and 47.9% of the men being 65 years old and over. Women had a lower educational level than men, and both had a lower educational level in the more aged stratum (**Table 1**). Most prevalent fracture risk factors were falls (20.3%), personal history of fracture (8.0%), and osteopenic diseases (12.3%) and, in general, risk factors were more prevalent with age. Using the Canadian thresholds (Papaioannou et al., 2010), 13.5% of the ESOSVAL population showed a high risk (≥3%) of hip fracture (0.4% in people under 65 years old and 29.4% in people of 65 and over). The proportion of the population at a high risk of hip fracture in people under 65 was 0.7% for women and 0.1% for men, while 22% of women and 1.7% of men from this age group were taking osteoporosis drugs and 20.6% of women and 2.4% of men were taking calcium and/or vitamin D supplements at recruitment.

**Figure 1:**
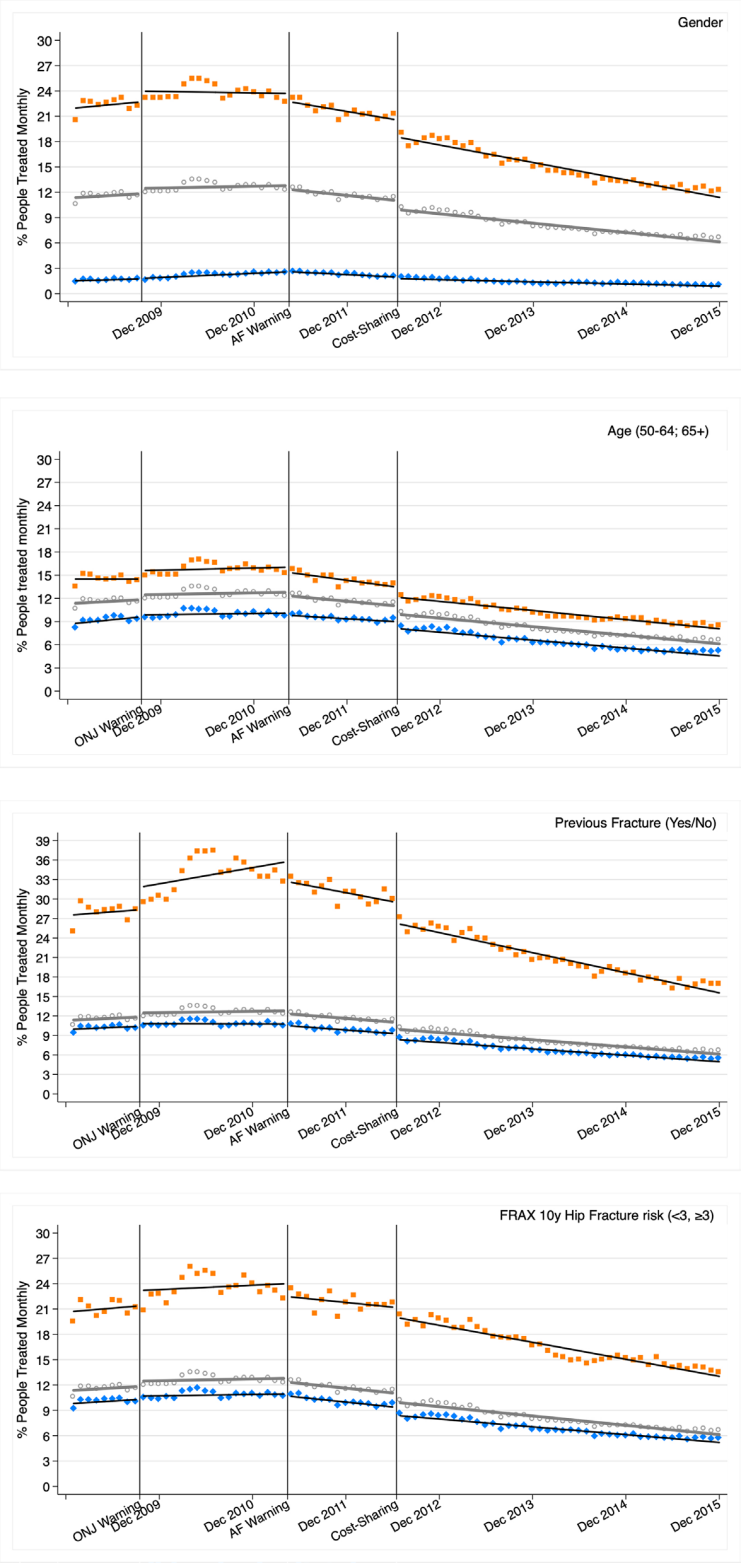
Osteoporosis treatment segmented linear regression trends 2009–2015 for all the ESOSVAL cohort and stratified by gender, age, previous fracture, and FRAX 10 years risk of hip fracture. ONJ: osteonecrosis jaw; AF: atypical fracture. Marks: circle (all); orange/square (women, ≥65 years, previous fracture, FRAX ≥3); blue/diamond (men, 50–64 years, no previous fracture; FRAX < 3). Lines represent the results of the regression, while marks (circles, squares, and diamonds) represent observations.

The percentage of people treated in the entire cohort grew from 10.6% of the cohort in Jan 2009 to a peak of 13.5% in May 2010, descending from that month to 6.7% in December 2015, a relative reduction of 59% from Jan 2009, and of 104% from the peak of treatment. **Figure 1** shows the results of the segmented linear regression models for the whole ESOSVAL cohort and stratified by gender, age, and previous fracture and FRAX 10-year risk of hip fracture. In all analyses, and despite the ONJ warming in Sept 2009, trends were rising until the AF warning in Apr 2011, starting a downward trend from that moment until the end of the period only altered by a sudden drop associated with the cost-sharing policy change in Jul 2012.

**Table 2** shows the most relevant parameters of the segmented regressions for the entire cohort and the stratum analyzed (see **Supplementary Materials for the complete models: Tables S2 to S6 and Figures S1 to S5**). In the entire ESOSVAL cohort, the proportion of people treated increased from an initial constant of 11.3% until the release of the AF warning when, with a non-significant immediate level change, a downward trend began with 0.11% fewer people treated each month. The change in the cost-sharing scheme abruptly reduced by 1.07% the proportion of people treated (immediate level change), but the downward trend initiated immediately after the AF warning was not affected. This pattern of downward trends associated with the AF warning and the level change associated with the cost-sharing change can be observed in all stratified analyses. Also, some of the higher consumption strata showed increases in the level change associated with the issue of the ONJ warning (women, 65 years and over, previous fracture, and FRAX risk of hip fracture ≥3%) and level changes associated with the issue of the AF warning (women, previous fracture, and FRAX risk of hip fracture ≥3%).

**Table 2:** Segmented regression parameters for all people, and stratified by gender, age, previous fracture, and FRAX 10 years risk of hip fracture.

	All	Gender		Age		Previous fracture		Hip FRAX ≥3%	
		Men	Women	50–64	65+	No	Yes	No	Yes
Initial constant	11.31*	1.50*	21.89*	8.66*	14.51*	9.92*	27.47*	9.76*	20.63*
Trend from start to ONJ warning	0.05	0.02	0.09	0.10*	−0.001	0.05	0.09	0.06	0.08
Constant 2nd period/ONJ warning issue	0.65*	0.67	1.31*	0.30	1.07*	0.43	3.42*	0.42	1.83*
Trend from ONJ warning to AF warning	−0.04	0.17	−0.10	−0.09	0.02	−0.05	0.12	−0.04	−0.04
Constant 3rd period/AT warning issue	−0.40	0.05	−0.90*	−0.24	−0.60	−0.20	−2.87*	−0.19	−1.47*
Trend from AF warning to cost-sharing change	−0.11*	−0.09*	−0.14*	−0.07*	−0.16*	−0.09*	−0.44*	−0.11*	−0.14
Constant 4th period/cost-sharing change	−1.07*	−0.20*	−2.02*	−0.87*	−1.32*	−0.87*	−3.21*	−0.97*	−1.14*
Trend from cost-sharing change	0.001	0.02*	−0.02	−0.03	0.04	0.01	−0.03	0.02	−0.07

ONJ, Osteonecrosis Jaw; AF, Atypical fracture.

n, 84 months; R^2^, from 0.93 to 0.98 according models. *p < 0.05.

**Figure 2:**
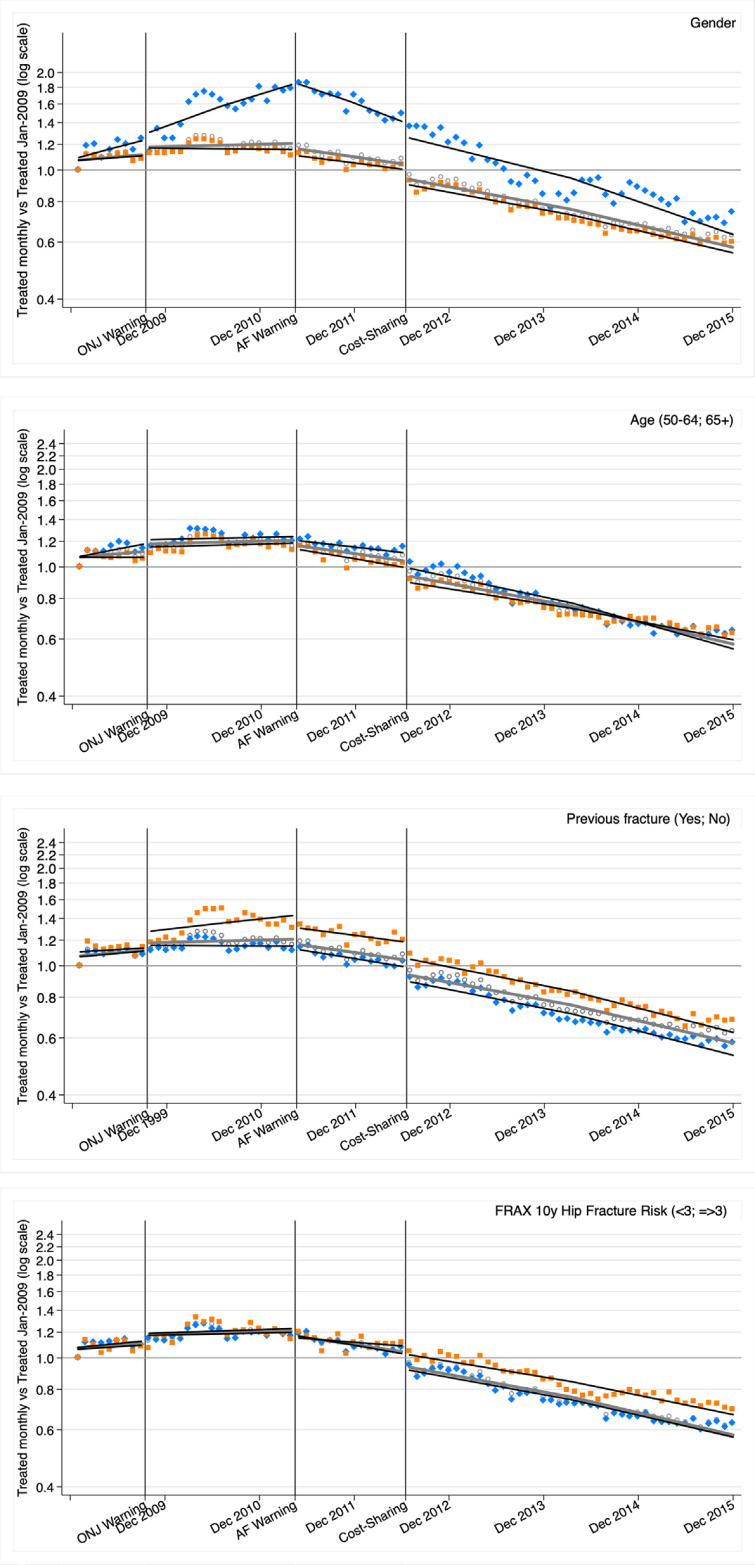
Ratio of osteoporosis treatment each month regarding January 2009. Segmented linear regression trends 2009–2015 for all the ESOSVAL cohort and stratified by gender, age, previous fracture, and FRAX 10 years risk of hip fracture. ONJ: osteonecrosis jaw; AF: atypical fracture. Marks: circle (all); orange/square (women, ≥65 years, previous fracture, FRAX ≥3); blue/diamond (men, 50–64 years, no previous fracture; FRAX < 3). Lines represent the results of the regression, while marks (circles, squares, and diamonds) represent observations.

**Figure 2** shows the segmented regressions with the ratio between the proportion of patients treated each month and the proportion treated in January 2009 (see **Supplementary Materials—Tables S7 to S11, Figures S6 to S10**—for model parameters). The downward trends initiated after the AF warning were similar for the different risk strata, somewhat more pronounced in men (who had previously experienced greater growth), although the relative decline at the end of the period was slightly lower in men and in people with a previous fracture (at the expense of a greater relative increase in the period prior to the AF warning), or with a FRAX hip fracture risk ≥3% (at the expense of a lower level change associated with the change in the cost-sharing scheme). By age, the reduction was similar in people both under and over 65 years old.

## Discussion

Our study shows that osteoporosis drug utilization in the Valencia region increased until mid-2011 and then started to decline, so that by the end of 2015 global consumption was around a half of 2009 and almost two thirds less than the maximum peak in 2010. The AF safety warning of April 2011 and to a lesser extent the increase in the pharmaceutical cost-sharing (associated with a sudden descent in the months immediately after July 2012 but without altering the temporary trend) seem to have had a strong influence on this decline, which nonetheless does not seem to be related to the clinical characteristics of patients, as we observe a similar relative decline in those with both a high and low risk of fracture. To the best of our knowledge, no previous studies in this field have assessed the impact of warnings on several risk strata (age, gender, risk of fracture).

The beginning of the decrease in the consumption of osteoporosis drugs happened at an earlier moment in Australia (Peeters et al., 2014), the UK (van der Velde et al., 2017), or the US (Jha et al., 2015; Balkhi et al., 2018), with a maximum peak in 2009 and starting to fall in 2010, coinciding with the FDA Warning on the association between long-term use of bisphosphonates and atypical fractures (requiring drug manufacturers to include a recommendation for considering discontinuation after 3–5 years of treatment in patients at a low risk of fracture). However, certain parallels exist as the Spanish Agency for Medicines and Medical Devices did not publish the warning on atypical fractures (simultaneously with the European Medicines Agency) until mid-2011, a year after the FDA warning. None of these previous studies in Australia, the UK, or the US evaluated the appropriateness of treatment according to patient risk factors, so these results cannot be compared with those of our study, but the decline of secondary prevention with osteoporosis drugs after hip fracture in the US started before 2010 intensified after the FDA 2010 warning (Kim et al., 2016; Desai et al., 2018). A cross-national study also seems to show a declining trend in bisphosphonate use following hip fracture after 2010 in Spain, the US, and Korea, compensated for in this last country by the use of other osteoporosis drugs (Kim et al., 2015).

In addition to bisphosphonate safety warnings issued by regulatory agencies, other factors may have contributed to the decline in the consumption of osteoporosis drugs in Spain. First the expiration of most patents, with the associated cessation of pharmaceutical promotion and proprietary firm efforts to neutralize the impact of warnings (note that warnings on jaw osteonecrosis with some bisphosphonate patents in force had little impact, if any, on osteoporosis drug utilization); second, the contagion from safety warnings on other osteoporosis drugs, including the practical withdrawal of calcitonin and strontium ranelate (see **Supplementary Material Table S1**); third, the influence from the previous FDA atypical fracture warning, with a wide repercussion in medical journals, scientific meetings and guidelines, including an important controversy about the suspension of the treatment and its duration (the so-called “therapeutic holidays”). And finally, and as studies in other therapeutic areas (González López-Valcárcel et al., 2017) and the results of our study show, the introduction of a new cost-sharing scheme with an 8–10% copayment for retired people (previously exempt) and increases in the copayment for most of the active working population and their families.

The benefit in terms of fracture prevention provided by bisphosphonates far outweighs the potential risks of atypical fracture and jaw osteonecrosis in most patients at a high risk of fracture (Maraka and Kennel, 2015; Hanley et al., 2017). Although our study does not directly address treatment appropriateness (or its absence), the analysis of fracture risk factors strongly suggests the existence of a high proportion of inappropriate treatment in low risk people (for instance, approximately three quarters of treatments in 2015 were dispensed to patients with FRAX 10-year risk of hip fracture below 3%) and also of a high proportion of inappropriate absence of treatment (only 14% of the ESOSVAL cohort patients with a 10-year risk of hip fracture equal to or above 3% were receiving treatment at the end of 2015). Therefore, and despite the decrease in osteoporosis drug consumption, a significant concern about overuse remains and is even reinforced with regard to underuse in patients at risk.

### Strengths and Limitations

Our study has strengths and limitations. Among the former, it should be noted that—even if introduced in the EMR—the baseline data was collected prospectively by doctors and nurses trained in osteoporosis and in the operational definitions of the study. Additionally, data from the VHS electronic prescription information system is of high quality, and includes paperless electronic prescription, the registration of any dispensation in any community pharmacy, and reimbursement to pharmacies in a traceable way for each pharmaceutical package and each patient.

Among the limitations, the first is the use of the baseline characteristic of the ESOSVAL cohort to stratify the risk of fracture, when several of these characteristics (e.g. the incidence of previous fracture or the FRAX scores) may have changed with advancing age in the 5–6 years of the cohort follow-up and the risk level of some patients could be misclassified in the final years of the study. Second, we have no information on zoledronic acid consumption, which is restricted to in hospital use in our country. Although it is likely that some patients may still be treated with this drug (thus our study would underestimate the proportion of patients treated), studies in other countries indicate that zoledronic acid has undergone a decrease in consumption similar to that of other bisphosphonates (Wysowski and Greene, 2013). Third, we have not analyzed the importance of the possible mechanisms operating in the decrease in osteoporosis drug consumption (non-adherence, discontinuation, therapeutic holidays, decrease of initiators or others), an essential aspect for the design of underuse improvement strategies or to assess the impact of this decrease on clinical outcomes, an essential element to establish the substantive importance of over and underuse. In any case, the current evidence would support a negative risk-benefit balance in the case of low-risk patients and positive in high-risk patients, with large gray areas in the intermediate risks and with respect to the duration of treatment or possible temporary discontinuations. Finally, doctors who enrolled patients in the ESOSVAL cohort were the object of an educational intervention coinciding with the cohort recruitment period (2009–2010), an aspect that could have modified the initial prescription behavior.

Despite these limitations, our study shows a worrying evolution of treatment for the prevention of osteoporotic fracture in our environment, where an important problem of overuse still remains, while the problem of underuse is intensified. This situation urgently requires approaches (professional and organizational) focused on high-risk population (especially in secondary prevention after hip and vertebral fracture) that selectively addresses underutilization, while continuing efforts to avoid treatments in low-risk people.

## Conclusion

The AEMPS ONJ warning of Sept 2009 was not associated with a decline in the consumption of osteoporosis drugs, while the AEMPS AF warning of Apr 2010 was associated with a significant decrease in the number of people treated, reinforced by the increase in the pharmaceutical cost-sharing occurred in 2012. As a result, in December 2015 only half of the patients that of May 2010 (the month with the highest proportion of treatment) were under treatment. Decreases in treatment affected patients both at a low and higher risk of fracture.

## Data Availability

The datasets for this study will not be made publicly available because legal restrictions on sharing the dataset apply as regulated by the Valencia regional government by means of legal resolution by the Valencia Health Agency [2009/13312] which forbids the cession of data to third parties (accessible at: http://www.san.gva.es/documents/152919/157920/resolucionsolicituddatos.pdf). Authorization to access the data may be requested to the Management Office of the Data Commission in the Valencia Health Agency (email: solicitud_datos@gva.es; telephone numbers: +34961-928207; +34 961-928198).

## Ethics Statement

All information relative to the patients was handled according to Spanish regulations on data protection and patients’ rights. All procedures performed in studies involving human participants were in accordance with the ethical standards of the institutional and/or national research committee and with the 1964 Helsinki Declaration and its later amendments or comparable ethical standards.

## Author Contributions

GS-G had full access to all the data in the study and takes responsibility for their integrity and of the accuracy of their analysis. GS-G, JS-G, IH-N, and SP were responsible for the study concept, design, and data acquisition. GS-G, IH-N, AG-S, and CR-B carried out the data preparation and the statistical analysis and AG-S drafted the manuscript. All authors participated in the analysis and interpretation of data and critical revision of the manuscript for important intellectual content. All approved the final version submitted for publication and agree to be accountable for all aspects of the work in ensuring that questions related to the accuracy or integrity of any part of the work are appropriately investigated and resolved.

## Funding

The ESOSVAL research program is co-funded by the Instituto de Salud Carlos III (grants PS09/02500, PI11/00238, and PI13/01721) from the Spanish Ministry of Health and the European Regional Development Fund, and by collaboration agreements established by FISABIO and the Valencia Department of Health with MSD Spain (2009–2012) and AMGEN S.A. (2010–2013), to conduct training and real-world research into musculoskeletal disorders and osteoporosis. None of the sponsors played any role in the design of the ESOSVAL studies, the collection, analysis or interpretation of data, the writing of the manuscript, or in the decision to submit it for publication.

## Conflict of Interest Statement

FISABIO has received research grants from various pharmaceutical companies (see funding of the ESOSVAL project).

The authors declare that the research was conducted in the absence of any commercial or financial relationships that could be construed as a potential conflict of interest.
