# Supplementary material for: Impact of Drug Safety Warnings and Cost-Sharing Policies on Osteoporosis Drug Utilization in Spain: A Major Reduction But With the Persistence of Over and Underuse. Data From the ESOSVAL Cohort From 2009 to 2015
Source: Front Pharmacol. 2019 Jul 10;10:768. doi: 10.3389/fphar.2019.00768 (PMC6635591; doi:10.3389/fphar.2019.00768)
Supplement: Supplementary file 1 [file Table_1.docx]

| Table S1. AEMPS warnings and informative notes on osteoporosis drugs (2009-2015) | |
| --- | --- |
| Nov 8, 2005 | Bisphosphonates for parenteral administration and jaw osteonecrosis. |
| Set, 2009 | Recommendations for the prevention of jaw osteonecrosis associated with treatment with bisphosphonates. |
| Apr, 2011 | Bisphosphonates and risk of atypical femur fractures. |
| Mar, 2012 | Strontium ranelate (Osseor®, Protelos®): risk of venous thromboembolism and serious dermatological reactions. New contraindications for use. |
| Jul, 2012 | Calcitonin: use restricted to short-term treatments. |
| Apr, 2013 | Calcitonin: suspension of the commercialization of intranasal preparations and restriction in the use of injectable preparations to short-term treatments. |
| Apr, 2013 | Strontium ranelate (Osseor®, Protelos®): risk of acute myocardial infarction |
| Jan, 2014 | Strontium ranelate (Osseor®, Protelos®): the European review concludes that the benefit-risk balance is unfavorable |
| Feb, 2014 | Completion of the review of the benefit-risk balance of strontium ranelate (Osseor®, Protelos®): restrictions in use. |
| Jul, 2014 | Strontium ranelate (Osseor®, Protelos®): qualified as a hospital diagnosis drug. |
| Set, 2014 | Denosumab (Prolia®, Xgeva®): risk of jaw osteonecrosis and hypocalcemia. |

| Table S2. Proportion of people treated (ESOSVAL Cohort). Segmented regression analysis. | | | | |
| --- | --- | --- | --- | --- |
|  | AGE: <65 AT RECRUITMENT | | | |
|  | Coef. | p | 95%CI | |
| Initial Constant | 11.31 | <0.001 | 10.78 | 11.85 |
| Trend from Start to ONJW | 0.05 | 0.260 | -0.04 | 0.15 |
| Constant 2nd period/ONJW | 0.65 | 0.026 | 0.08 | 1.22 |
| Trend from ONJW to AFW | -0.04 | 0.466 | -0.93 | 0.06 |
| Constant 3rd period/AFW | -0.40 | 0.131 | -0.93 | 0.12 |
| Trend from AFW to Cost-sharing change | -0.11 | <0.001 | -0.17 | -0.05 |
| Constant 4th period/Cost-sharing change | -1.07 | <0.001 | -1.51 | -0.63 |
| Trend from Cost-Sharing change | <0.01 | 0.930 | -0.05 | 0.05 |
| n=84 months; R^2^: 0.976. ONJW: Osteonecrosis Jaw Warning; AFW: Atypical femur Fracture Warning | | | | |
|  | | | | |
| Figure S1. Segmented linear regression. Entire ESOSVAL Cohort | | | | |
| 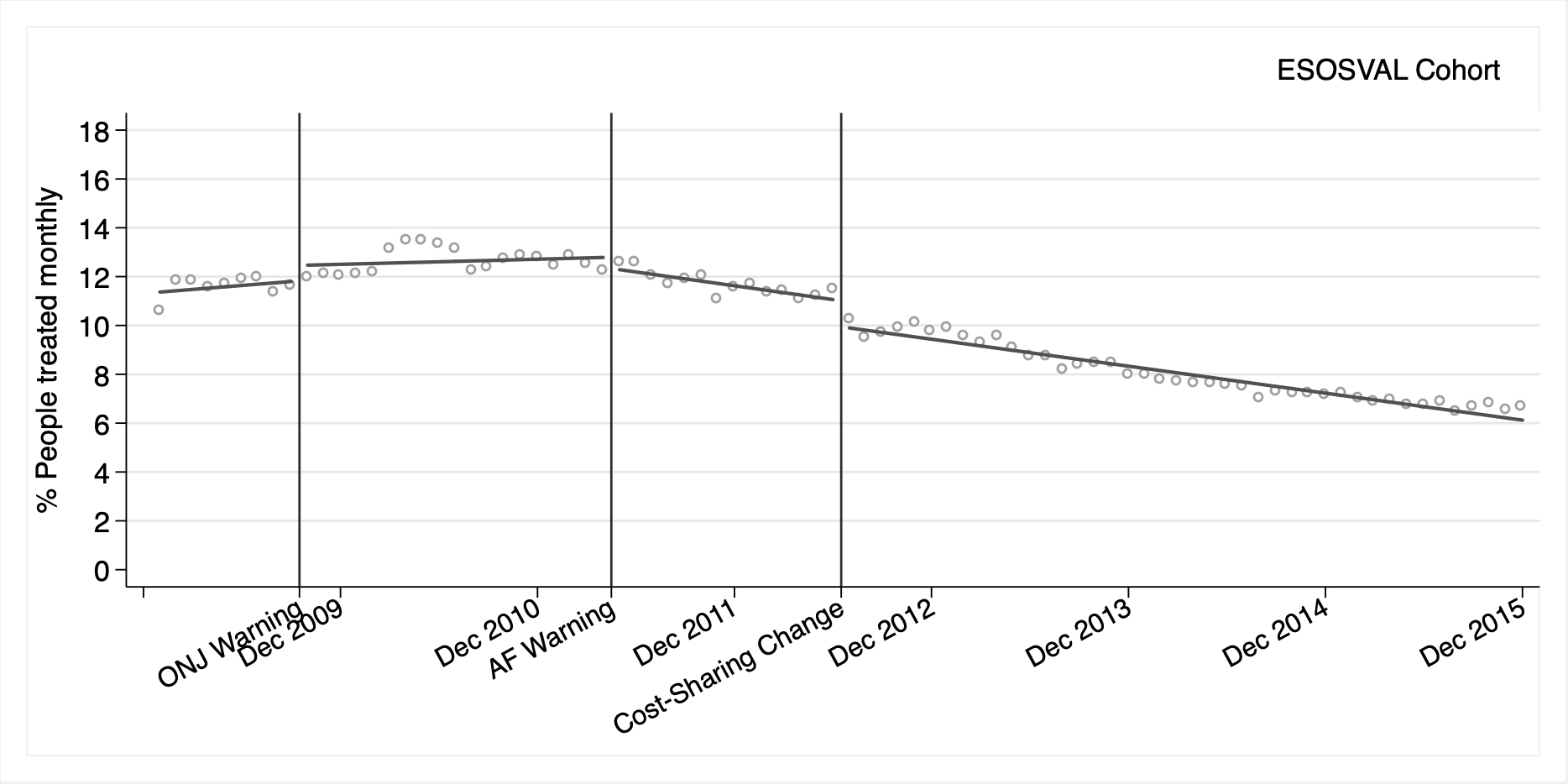 | | | | |

| Table S3. Proportion of people treated by gender. Segmented regression analysis. | | | | | | | | | |
| --- | --- | --- | --- | --- | --- | --- | --- | --- | --- |
|  | WOMEN | | | |  | MEN | | | |
|  | Coef. | p | 95%CI | |  | Coef. | p | 95%CI | |
| Initial Constant | 21.89 | <0.001 | 20.93 | 22.85 |  | 1.50 | <0.001 | 1.31 | 1.69 |
| Trend from Start to ONJW | 0.09 | 0.317 | -0.08 | 0.26 |  | 0.02 | 0.156 | -0.01 | 0.06 |
| Constant 2nd period/ONJW | 1.31 | 0.013 | 0.28 | 2.33 |  | 0.07 | 0.514 | -0.16 | 0.27 |
| Trend from ONJW to AFW | -0.10 | 0.267 | -0.28 | -0.08 |  | 0.02 | 0.352 | -0.02 | 0.05 |
| Constant 3rd period/AFW | -0.90 | 0.062 | -1.85 | 0.04 |  | 0.05 | 0.586 | -0.13 | 0.24 |
| Trend from AFW to Cost-sharing change | -0.14 | 0.008 | -0.24 | -0.04 |  | -0.09 | <0.001 | -0.11 | -0.07 |
| Constant 4th period/Cost-sharing change | -2.02 | <0.001 | -2.81 | -1.23 |  | -0.20 | 0.013 | -0.35 | -0.04 |
| Trend from Cost-Sharing change | -0.02 | 0.714 | -0.11 | 0.07 |  | 0.02 | 0.007 | 0.01 | 0.04 |
| n=84 months; R^2^: 0.932 (men); 0.977 (Women). ONJW: Osteonecrosis Jaw Warning; AFW: Atypical femur Fracture Warning | | | | | | | | | |
|  | | | | | | | | | |
| Figure S2. Segmented linear regression by gender | | | | | | | | | |
| 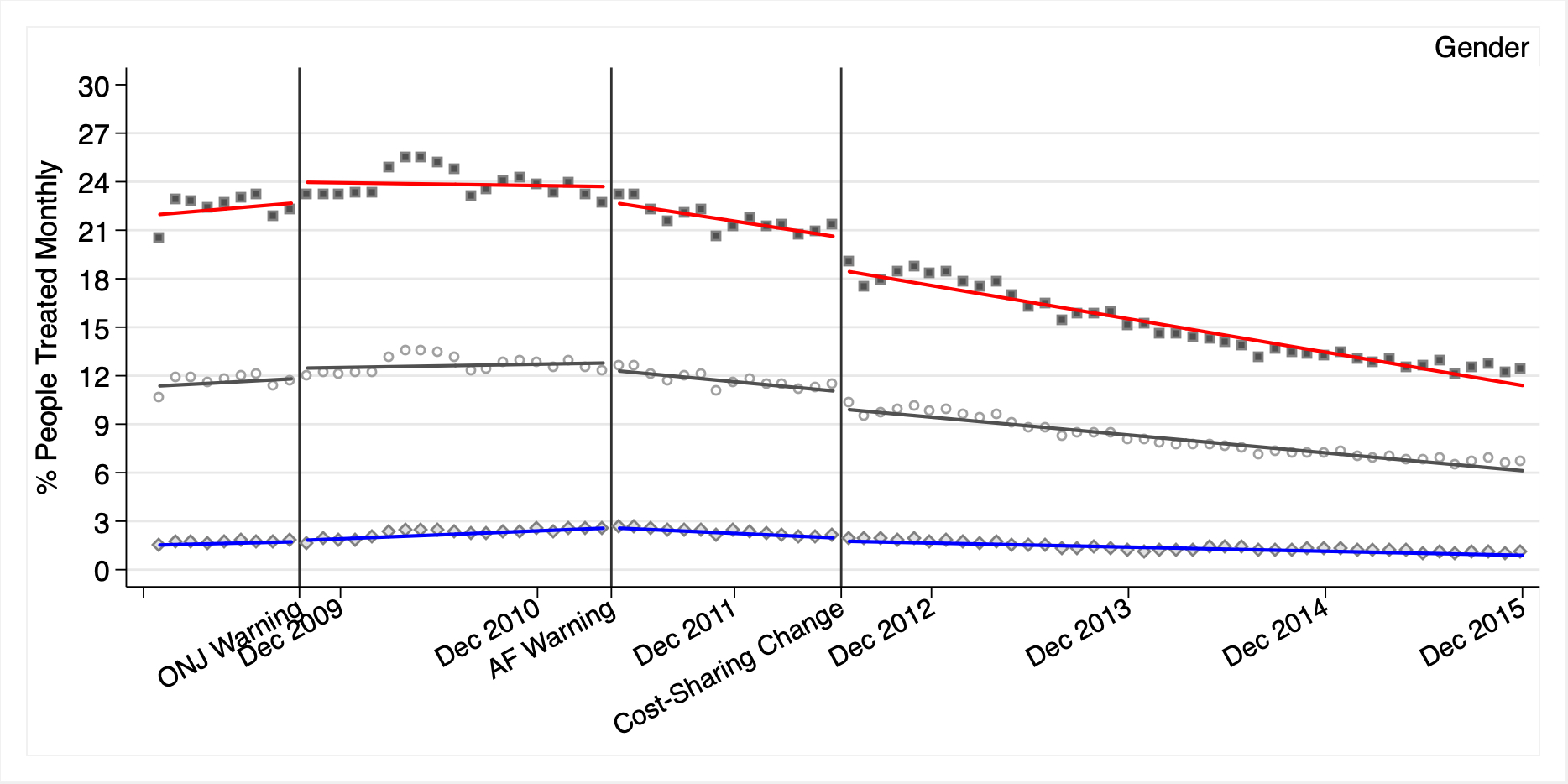 | | | | | | | | | |
| Women: red line; Men: blue line; All: grey line. ONJ: Osteonecrosis Jaw; AF: Atypical fracture | | | | | | | | | |

| Table S4. Proportion of people treated by age. Segmented regression analysis. | | | | | | | | | |
| --- | --- | --- | --- | --- | --- | --- | --- | --- | --- |
|  | 50-64 years old | | | |  | 65 years and over | | | |
|  | Coef. | p | 95%CI | |  | Coef. | p | 95%CI | |
| Initial Constant | 8.66 | <0.001 | 8.18 | 9.14 |  | 14.51 | <0.001 | 13.83 | 15.18 |
| Trend from Start to ONJW | 0.10 | 0.024 | 0.01 | 0.19 |  | <-0.01 | 0.991 | -0.12 | 0.12 |
| Constant 2nd period/ONJW | 0.30 | 0.248 | -0.21 | 0.82 |  | 1.07 | 0.004 | 0.35 | 1.80 |
| Trend from ONJW to AFW | -0.09 | 0.057 | -0.18 | <0.01 |  | 0.02 | 0.699 | -0.10 | 0.15 |
| Constant 3rd period/AFW | -0.24 | 0.313 | -0.72 | 0.23 |  | -0.60 | 0.074 | -1.23 | 0.06 |
| Trend from AFW to Cost-sharing change | -0.07 | 0.008 | -0.12 | -0.02 |  | -0.16 | <0.001 | -0.23 | -0.09 |
| Constant 4th period/Cost-sharing change | -0.87 | <0.001 | -1.27 | -0.47 |  | -1.32 | <0.001 | -1.87 | -0.77 |
| Trend from Cost-Sharing change | -0.02 | 0.268 | -0.07 | 0.02 |  | 0.04 | 0.233 | -0.02 | 0.10 |
| n=84 months; R^2^: 0.973 (65y and over); 0.968 (50-64y). ONJW: Osteonecrosis Jaw Warning; AFW: Atypical femur Fracture Warning | | | | | | | | | |
|  | | | | | | | | | |
| Figure S3. Segmented linear regression by age | | | | | | | | | |
| 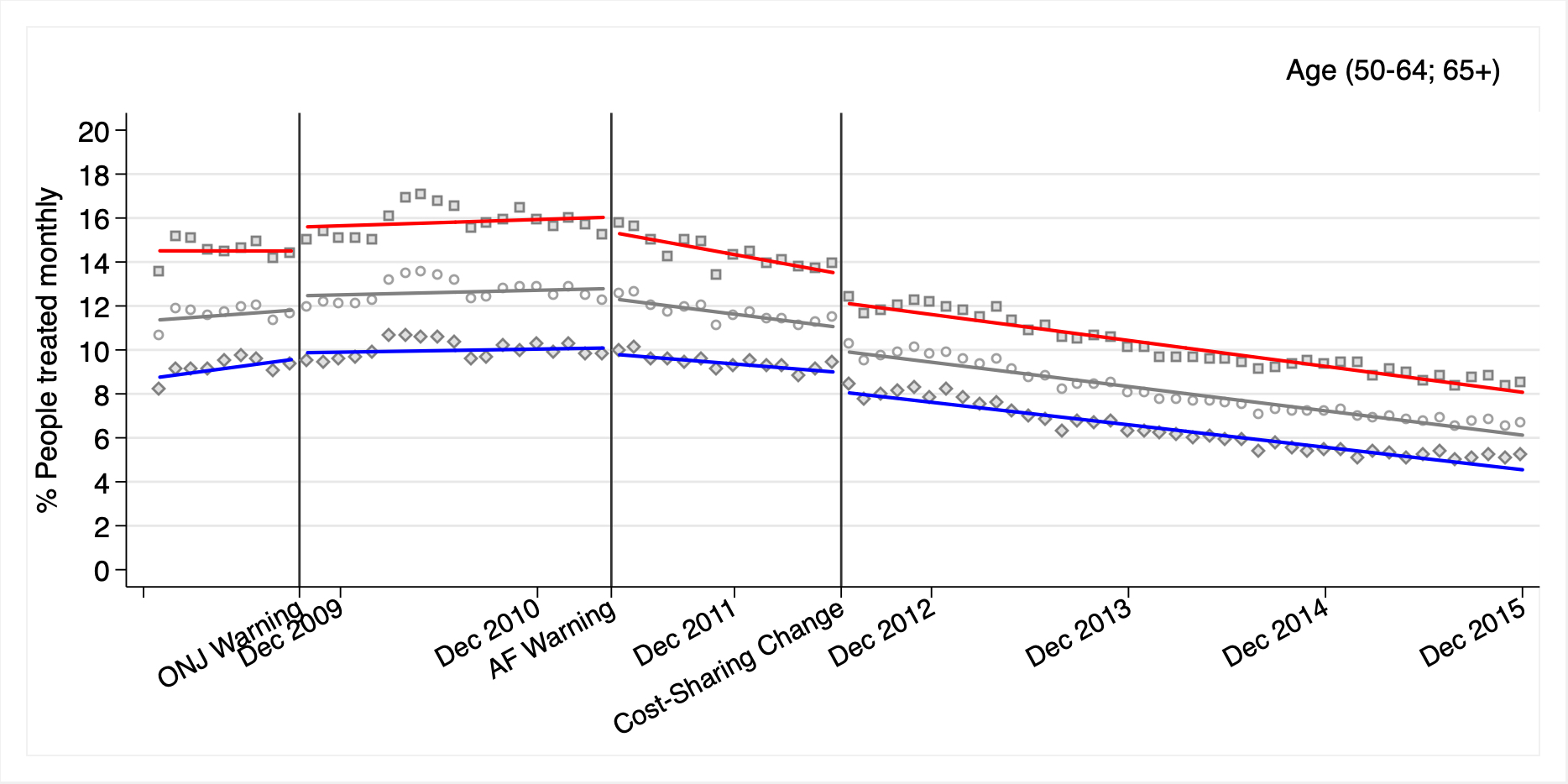 | | | | | | | | | |
| 65y and over: red line; 50-64y: blue line; All: grey line. ONJ: Osteonecrosis Jaw; AF: Atypical fracture | | | | | | | | | |

| Table S5. Proportion of people treated by antecedent of previous fracture. Segmented regression analysis. | | | | | | | | | |
| --- | --- | --- | --- | --- | --- | --- | --- | --- | --- |
|  | No previous fracture | | | |  | Previous fracture | | | |
|  | Coef. | p | 95%CI | |  | Coef. | p | 95%CI | |
| Initial Constant | 9.92 | <0.001 | 9.48 | 10.36 |  | 27.47 | <0.001 | 25.44 | 29.50 |
| Trend from Start to ONJW | 0.05 | 0.201 | -0.03 | 0.13 |  | 0.09 | 0.616 | -0.27 | 0.45 |
| Constant 2nd period/ONJW | 0.43 | 0.075 | -0.04 | 0.90 |  | 3.42 | 0.002 | 1.25 | 5.60 |
| Trend from ONJW to AFW | -0.05 | 0.202 | -0.14 | 0.03 |  | 0.12 | 0.542 | -0.26 | 0.49 |
| Constant 3rd period/AFW | -0.20 | 0.354 | 0.64 | 0.23 |  | -2.87 | 0.006 | -4.87 | -0.86 |
| Trend from AFW to Cost-sharing change | -0.09 | <0.001 | -0.14 | 0.04 |  | -0.44 | <0.001 | -0.66 | -0.22 |
| Constant 4th period/Cost-sharing change | -0.87 | <0.001 | -1.24 | -0.51 |  | -3.22 | <0.001 | -4.88 | -1.55 |
| Trend from Cost-Sharing change | 0.01 | 0.714 | -0.03 | 0.05 |  | -0.03 | 0.762 | -0.22 | 0.15 |
| n=84 months; R^2^: 0.954 (Previous fracture); 0.979 (No previous fracture). ONJW: Osteonecrosis Jaw Warning; AFW: Atypical femur Fracture Warning | | | | | | | | | |
|  | | | | | | | | | |
| Figure S4. Segmented linear regression by previous fracture antecedent | | | | | | | | | |
| 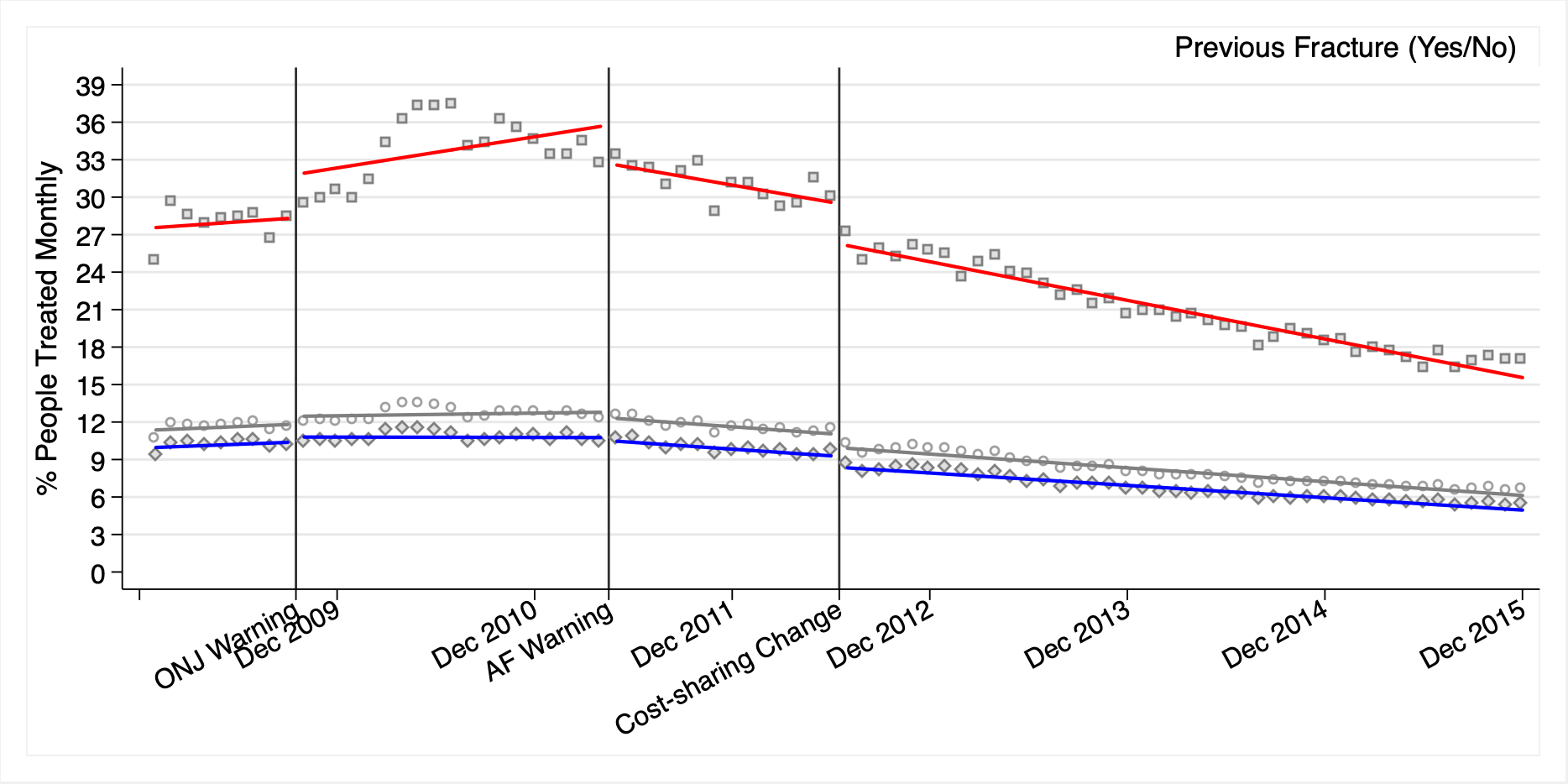 | | | | | | | | | |
| Yes: red line; 50-64y: No; All: grey line. ONJ: Osteonecrosis Jaw; AF: Atypical fracture | | | | | | | | | |

| Table S6. Proportion of people treated by FRAX risk of hip fracture. Segmented regression analysis. | | | | | | | | | |
| --- | --- | --- | --- | --- | --- | --- | --- | --- | --- |
|  | <3% | | | |  | ≥3% | | | |
|  | Coef. | p | 95%CI | |  | Coef. | p | 95%CI | |
| Initial Constant | 9.76 | <0.001 | 9.30 | 10.21 |  | 20.63 | <0.001 | 19.28 | 21.97 |
| Trend from Start to ONJW | 0.06 | 0.171 | -0.02 | 0.14 |  | 0.08 | 0.514 | -0.16 | 0.32 |
| Constant 2nd period/ONJW | 0.42 | 0.092 | -0.07 | 0.91 |  | 1.83 | 0.013 | 0.39 | 3.27 |
| Trend from ONJW to AFW | -0.04 | 0.312 | -0.13 | 0.04 |  | -0.04 | 0.779 | -0.29 | 0.22 |
| Constant 3rd period/AFW | -0.19 | 0.410 | -0.64 | 0.26 |  | -1.47 | 0.030 | -2.80 | -0.14 |
| Trend from AFW to Cost-sharing change | -0.11 | <0.001 | -0.16 | -0.06 |  | -0.14 | 0.067 | -0.28 | 0.01 |
| Constant 4th period/Cost-sharing change | -0.97 | <0.001 | -1.35 | -0.60 |  | -1.14 | 0.043 | -2.25 | -0.04 |
| Trend from Cost-Sharing change | -0.02 | 0.392 | -0.02 | 0.06 |  | -0.08 | 0.233 | -0.20 | 0.05 |
| n=84 months; R^2^: 0.977 (<3%); 0.931 (≥3%). ONJW: Osteonecrosis Jaw Warning; AFW: Atypical femur Fracture Warning | | | | | | | | | |
|  | | | | | | | | | |
| Figure S5. Segmented linear regression by FRAX risk of hip fracture. | | | | | | | | | |
| 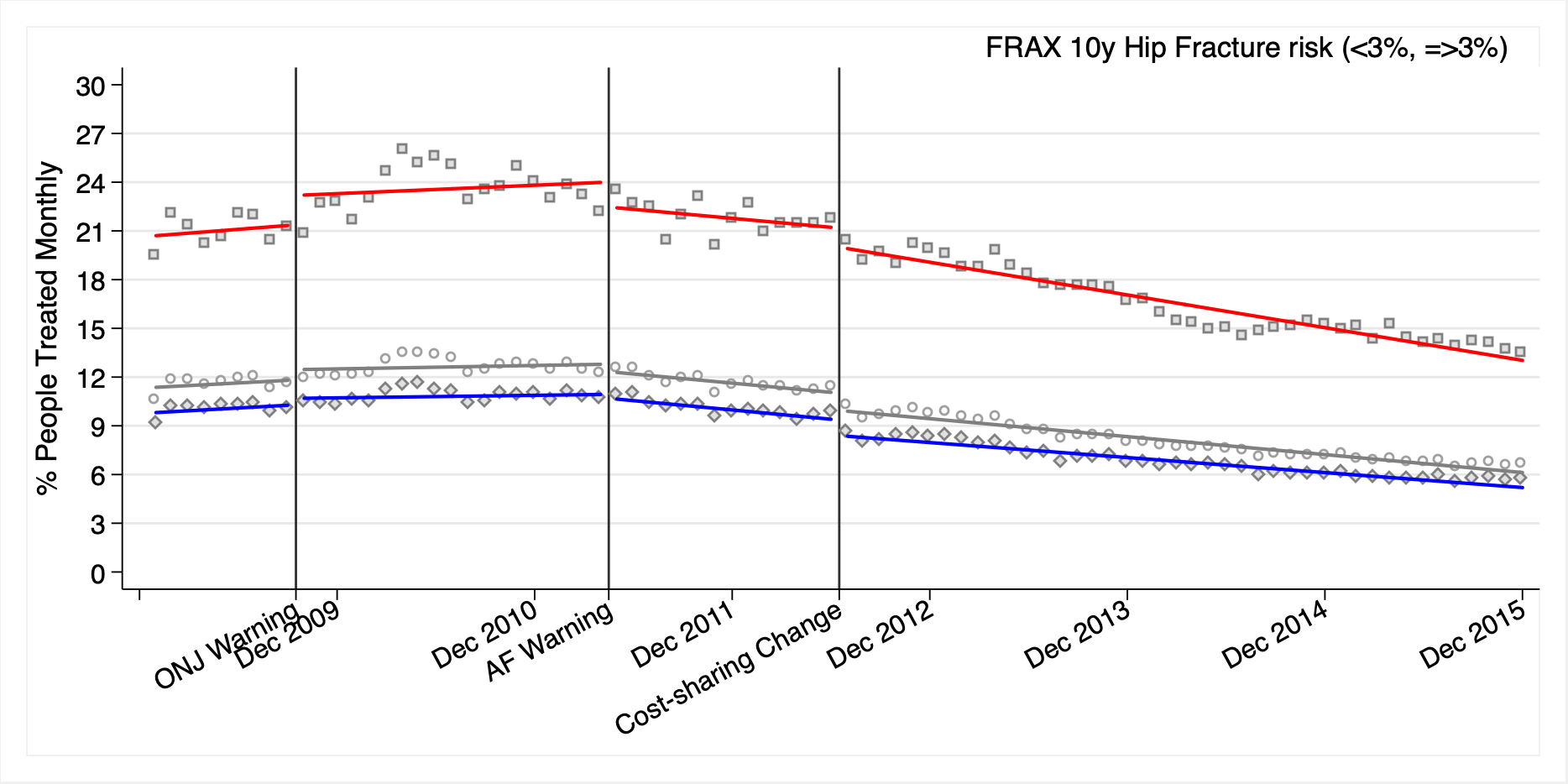 | | | | | | | | | |
| ≥3%: red line; <3%: blue line; All: grey line. ONJ: Osteonecrosis Jaw; AF: Atypical fracture | | | | | | | | | |

| Table S7. Ratio of monthly osteoporosis treatment regarding January 2009. Segmented regression analysis for the entire ESOSVAL cohort. | | | | |
| --- | --- | --- | --- | --- |
|  | AGE: <65 AT RECRUITMENT | | | |
|  | Coef. | p | 95%CI | |
| Initial Constant | 1.069 | <0.001 | 1.019 | 1.119 |
| Trend from Start to ONJW | 0.005 | 0.260 | -0.004 | 0.014 |
| Constant 2nd period/ONJW | 0.061 | 0.026 | 0.007 | 0.116 |
| Trend from ONJW to AFW | -0.003 | 0.466 | -0.013 | 0.006 |
| Constant 3rd period/AFW | -0.038 | 0.131 | -0.088 | 0.011 |
| Trend from AFW to Cost-sharing change | -0.011 | <0.001 | -0.016 | -0.005 |
| Constant 4th period/Cost-sharing change | -0.101 | <0.001 | -0.142 | -0.060 |
| Trend from Cost-Sharing change | <0.001 | 0.930 | -0.004 | 0.004 |
| n=84 months; R^2^: 0.976. ONJW: Osteonecrosis Jaw Warning; AFW: Atypical femur Fracture Warning | | | | |
|  | | | | |
| Figure S6. Ratio monthly treatment / Jan 2009. Segmented linear regression. | | | | |
| 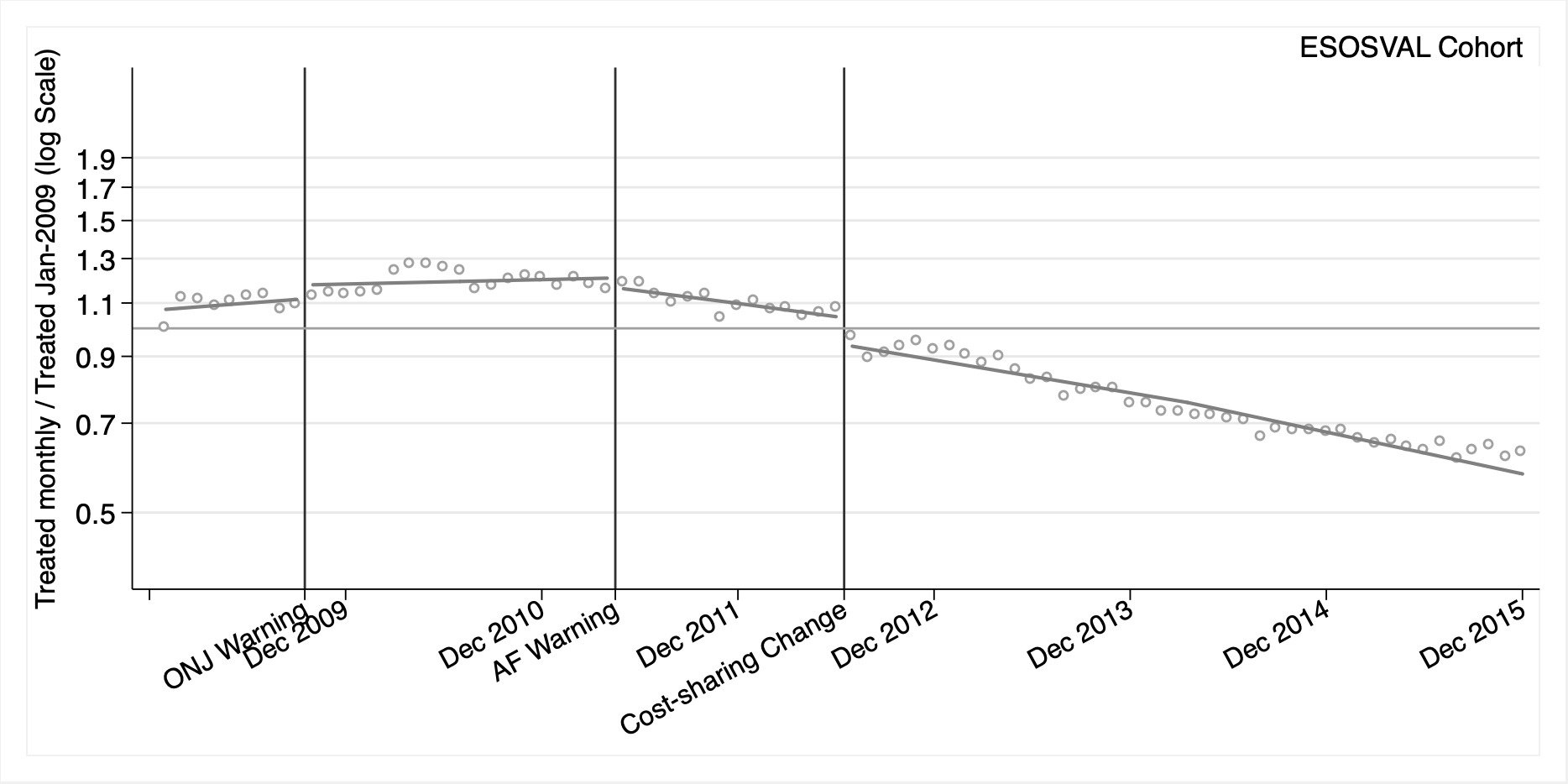 | | | | |

| Table S8. Ratio of monthly osteoporosis treatment regarding January 2009. Segmented regression analysis stratified by gender. | | | | | | | | | |
| --- | --- | --- | --- | --- | --- | --- | --- | --- | --- |
|  | MEN | | | |  | WOMEN | | | |
|  | Coef. | p | 95%CI | |  | Coef. | p | 95%CI | |
| Initial Constant | 1.076 | <0.001 | 0.941 | 1.211 |  | 1.068 | <0.001 | 1.022 | 1.115 |
| Trend from Start to ONJW | 0.017 | 0.156 | -0.007 | 0.041 |  | 0.004 | 0.317 | -0.004 | 0.012 |
| Constant 2nd period/ONJW | 0.478 | 0.514 | -0.097 | 0.193 |  | 0.064 | 0.013 | 0.013 | 0.114 |
| Trend from ONJW to AFW | 0.012 | 0.352 | -0.013 | 0.037 |  | -0.005 | 0.267 | -0.14 | 0.004 |
| Constant 3rd period/AFW | 0.037 | 0.586 | -0.097 | 0.170 |  | -0.044 | 0.062 | -0.090 | 0.002 |
| Trend from AFW to Cost-sharing change | -0.062 | <0.001 | -0.076 | -0.047 |  | -0.007 | 0.008 | -0.012 | -0.002 |
| Constant 4th period/Cost-sharing change | -0.142 | 0.013 | -0.253 | -0.031 |  | -0.099 | <0.001 | -0.137 | -0.060 |
| Trend from Cost-Sharing change | 0.018 | 0.007 | 0.005 | 0.030 |  | -0.001 | 0.714 | -0.005 | 0.003 |
| n=84 months; R^2^: 0.932 (men); 0.979 (Women). ONJW: Osteonecrosis Jaw Warning; AFW: Atypical femur Fracture Warning | | | | | | | | | |
|  | | | | | | | | | |
| Figure S7. Segmented linear regression by gender | | | | | | | | | |
| 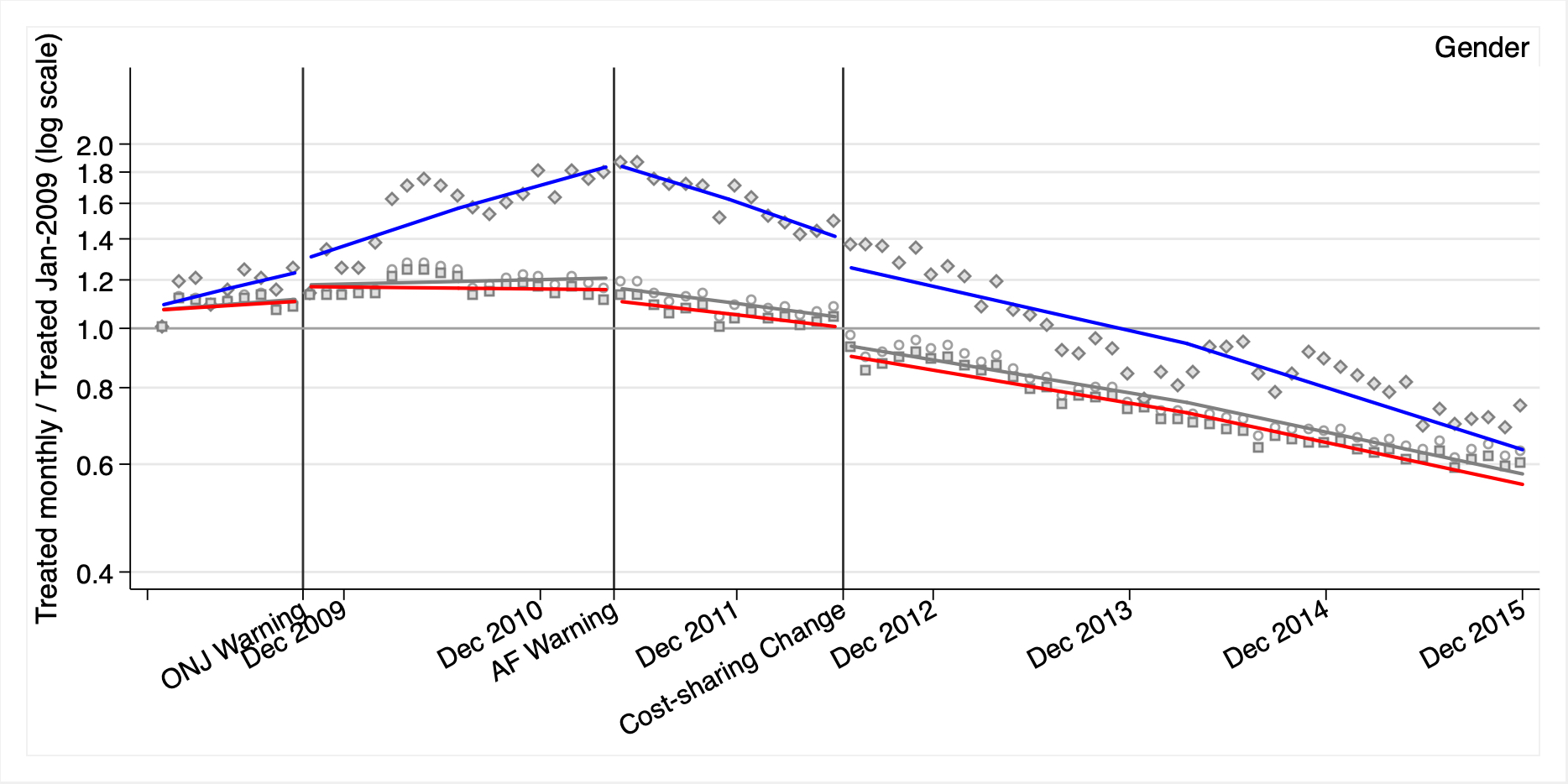 | | | | | | | | | |
| Women: red line; Men: blue line; All: grey line. ONJ: Osteonecrosis Jaw; AF: Atypical fracture | | | | | | | | | |

| Table S9. Ratio of monthly osteoporosis treatment regarding January 2009. Segmented regression analysis stratified by age. | | | | | | | | | |
| --- | --- | --- | --- | --- | --- | --- | --- | --- | --- |
|  | 50-64 years old | | | |  | 65 years and over | | | |
|  | Coef. | p | 95%CI | |  | Coef. | p | 95%CI | |
| Initial Constant | 1.065 | <0.001 | 1.006 | 1.125 |  | 1.072 | <0.001 | 1.022 | 1.121 |
| Trend from Start to ONJW | 0.012 | 0.024 | 0.002 | 0.023 |  | <0.001 | 0.991 | -0.009 | 0.009 |
| Constant 2nd period/ONJW | 0.037 | 0.248 | -0.026 | 0.101 |  | 0.079 | 0.004 | 0.026 | 0.133 |
| Trend from ONJW to AFW | -0.011 | 0.057 | -0.022 | <0.001 |  | 0.002 | 0.699 | -0.007 | 0.011 |
| Constant 3rd period/AFW | -0.030 | 0.313 | -0.089 | 0.029 |  | -0.045 | 0.074 | -0.093 | 0.004 |
| Trend from AFW to Cost-sharing change | -0.009 | 0.008 | -0.015 | -0.002 |  | -0.012 | <0.001 | -0.017 | -0.006 |
| Constant 4th period/Cost-sharing change | -0.107 | <0.001 | -0.156 | -0.058 |  | -0.097 | <0.001 | -0.138 | -0.057 |
| Trend from Cost-Sharing change | -0.003 | 0.268 | -0.009 | 0.002 |  | 0.003 | 0.233 | -0.002 | 0.007 |
| n=84 months; R^2^: 0.971 (65y and over); 0.971(50-64y). ONJW: Osteonecrosis Jaw Warning; AFW: Atypical femur Fracture Warning | | | | | | | | | |
|  | | | | | | | | | |
| Figure S8. Segmented linear regression by age | | | | | | | | | |
| 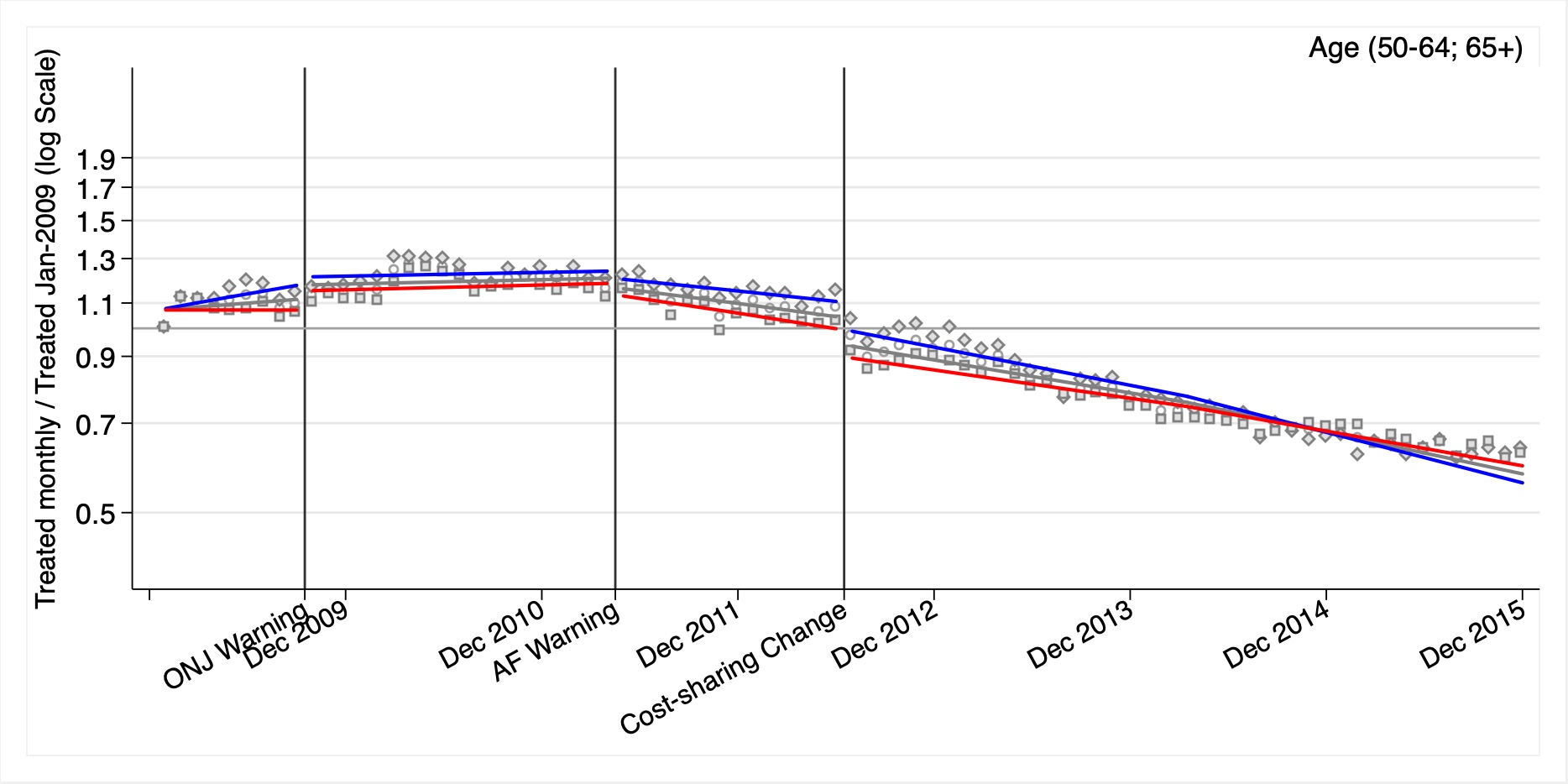 | | | | | | | | | |
| 65y and over: red line; 50-64y: blue line; All: grey line. ONJ: Osteonecrosis Jaw; AF: Atypical fracture | | | | | | | | | |

.

| Table S10. Proportion of people treated by antecedent of previous fracture. Segmented regression analysis. | | | | | | | | | |
| --- | --- | --- | --- | --- | --- | --- | --- | --- | --- |
|  | No previous fracture | | | |  | Previous fracture | | | |
|  | Coef. | p | 95%CI | |  | Coef. | p | 95%CI | |
| Initial Constant | 1.061 | <0.001 | 1.014 | 1.109 |  | 1.101 | <0.001 | 1.020 | 1.183 |
| Trend from Start to ONJW | 0.005 | 0.201 | -0.003 | 0.014 |  | 0.004 | 0.616 | -0.011 | 0.018 |
| Constant 2nd period/ONJW | 0.046 | 0.075 | -0.005 | 0.097 |  | 0.137 | 0.002 | 0.050 | 0.224 |
| Trend from ONJW to AFW | -0.006 | 0.202 | -0.014 | 0.003 |  | 0.005 | 0.542 | -0.010 | 0.020 |
| Constant 3rd period/AFW | -0.022 | 0.354 | -0.068 | 0.025 |  | -0.115 | 0.006 | -0.195 | -0.035 |
| Trend from AFW to Cost-sharing change | -0.009 | <0.001 | -0.014 | -0.004 |  | -0.017 | <0.001 | -0.026 | -0.009 |
| Constant 4th period/Cost-sharing change | -0.093 | <0.001 | -0.132 | -0.055 |  | -0.129 | <0.001 | -0.196 | -0.062 |
| Trend from Cost-Sharing change | 0.001 | 0.714 | -0.004 | 0.005 |  | -0.001 | 0.762 | -0.009 | 0.006 |
| n=84 months; R^2^: 0.954 (Previous fracture); 0.979 (No previous fracture). ONJW: Osteonecrosis Jaw Warning; AFW: Atypical femur Fracture Warning | | | | | | | | | |
|  | | | | | | | | | |
| Figure S9. Segmented linear regression by previous fracture antecedent | | | | | | | | | |
| 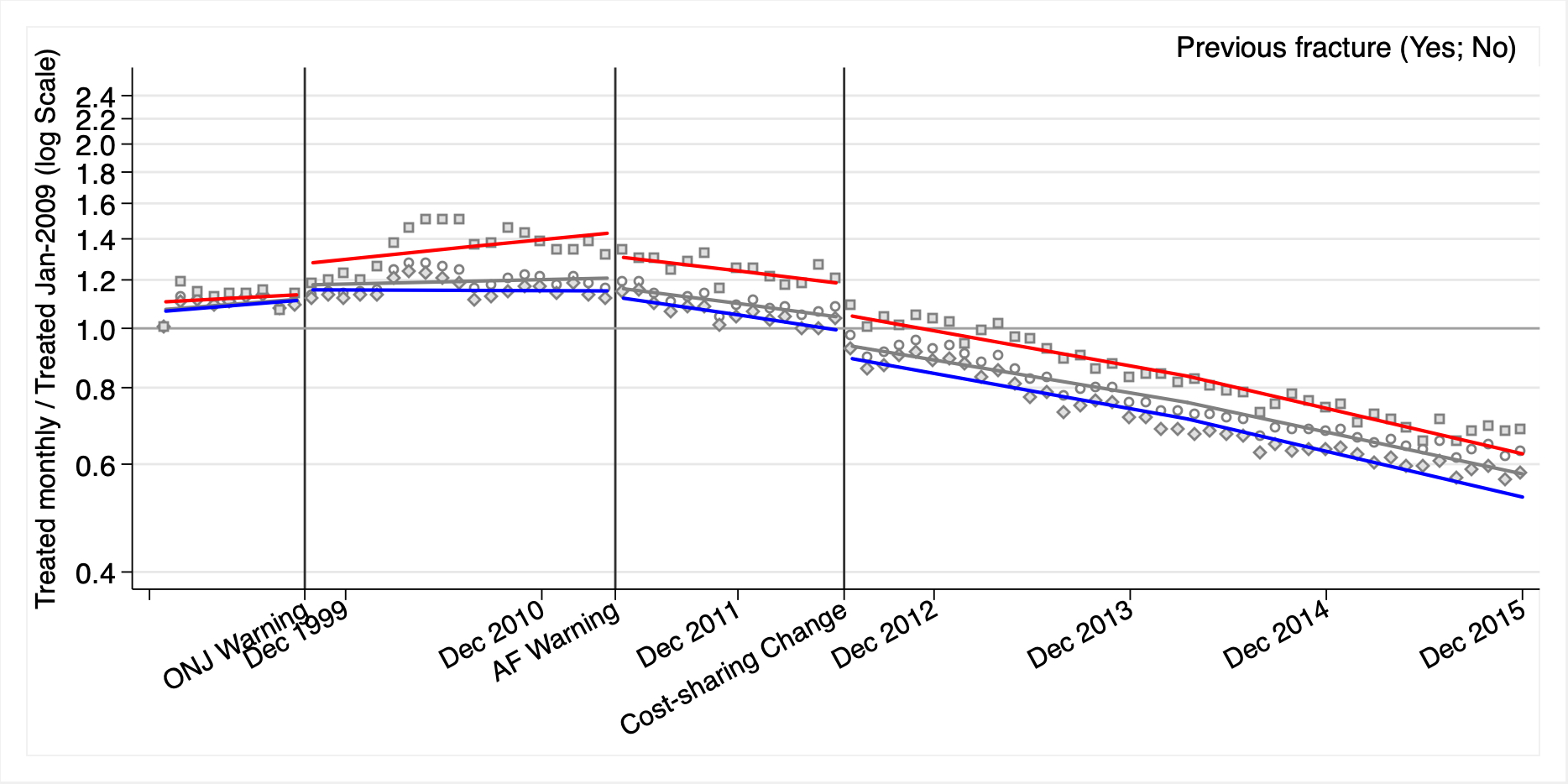 | | | | | | | | | |
| Yes: red line; 50-64y: No; All: grey line. ONJ: Osteonecrosis Jaw; AF: Atypical fracture | | | | | | | | | |

| Table S11. Proportion of people treated by FRAX risk of hip fracture. Segmented regression analysis. | | | | | | | | | |
| --- | --- | --- | --- | --- | --- | --- | --- | --- | --- |
|  | <3% | | | |  | ≥3% | | | |
|  | Coef. | p | 95%CI | |  | Coef. | p | 95%CI | |
| Initial Constant | 1.069 | <0.001 | 1.019 | 1.119 |  | 1.058 | <0.001 | 0.989 | 1.127 |
| Trend from Start to ONJW | 0.006 | 0.171 | -0.003 | 0.015 |  | 0.004 | 0.514 | -0.008 | 0.016 |
| Constant 2nd period/ONJW | 0.046 | 0.092 | -0.008 | 0.099 |  | 0.094 | 0.013 | 0.020 | 0.168 |
| Trend from ONJW to AFW | -0.005 | 0.312 | -0.014 | 0.005 |  | -0.002 | -0.779 | -0.015 | 0.011 |
| Constant 3rd period/AFW | -0.020 | 0.410 | -0.070 | 0.029 |  | -0.075 | -0.030 | -0.143 | -0.007 |
| Trend from AFW to Cost-sharing change | -0.012 | <0.001 | -0.017 | -0.006 |  | -0.007 | -0.067 | -0.014 | 0.001 |
| Constant 4th period/Cost-sharing change | -0.107 | <0.001 | -0.148 | -0.066 |  | -0.058 | -0.043 | -0.115 | -0.002 |
| Trend from Cost-Sharing change | 0.002 | 0.392 | -0.003 | 0.007 |  | -0.004 | -0.233 | -0.010 | 0.003 |
| n=84 months; R^2^: 0.977 (<3%); 0.937 (≥3%). ONJW: Osteonecrosis Jaw Warning; AFW: Atypical femur Fracture Warning | | | | | | | | | |
|  | | | | | | | | | |
| Figure S10. Segmented linear regression by FRAX risk of hip fracture. | | | | | | | | | |
| 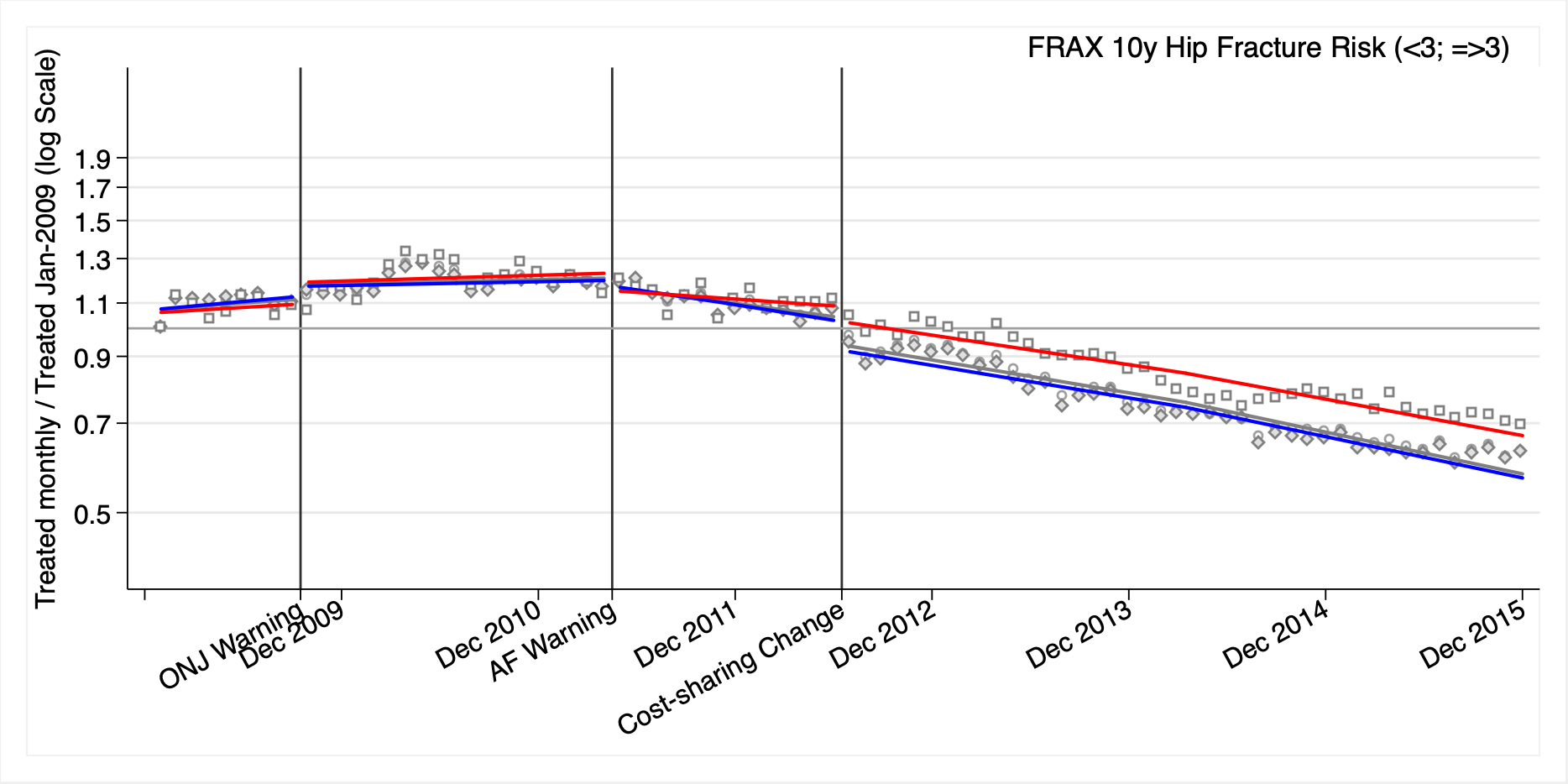 | | | | | | | | | |
| ≥3%: red line; <3%: blue line; All: grey line. ONJ: Osteonecrosis Jaw; AF: Atypical fracture | | | | | | | | | |

| Table S12. Annual consumption (months of treatment) of osteoporosis drugs, ratio to 2009 and market share in the ESOSVAL cohort (2009-2016) | | | | | | | |
| --- | --- | --- | --- | --- | --- | --- | --- |
|  | 2009 | 2010 | 2011 | 2012 | 2013 | 2014 | 2015 |
| Total |  |  |  |  |  |  |  |
| Months treat (n) | 15,487 | 16,941 | 15,846 | 13,715 | 11,282 | 9,379 | 8,375 |
| Ratio to 2009 | 1.00 | 1.09 | 1.02 | 0.89 | 0.73 | 0.61 | 0.54 |
| Bisphosphonates alone | | | | | | | |
| Months treat (n) | 9,588 | 10,685 | 9,642 | 7,911 | 6,288 | 4,882 | 4,030 |
| Ratio to 2009 | 1.00 | 1.11 | 1.01 | 0.83 | 0.66 | 0.51 | 0.42 |
| Market Share (%) | 61.91 | 63.07 | 60.85 | 57.68 | 55.73 | 52.05 | 48.12 |
| Bisphosphonates in combination | | | | | | | |
| Months treat (n) | 2,318 | 2,964 | 2,824 | 2,438 | 2,042 | 1,783 | 1,511 |
| Ratio to 2009 | 1.00 | 1.28 | 1.22 | 1.05 | 0.88 | 0.77 | 0.65 |
| Market Share (%) | 14.97 | 17.50 | 17.82 | 17.78 | 18.10 | 19.01 | 18.04 |
| Raloxifen | | | | | | | |
| Months treat (n) | 1,760 | 1,446 | 1,445 | 1,477 | 1,265 | 1,100 | 1,012 |
| Ratio to 2009 | 1.00 | 0.82 | 0.82 | 0.84 | 0.72 | 0.63 | 0.58 |
| Market Share (%) | 11.36 | 8.54 | 9.12 | 10.77 | 11.21 | 11.73 | 12.08 |
| Calcitonins | | | | | | | |
| Months treat (n) | 460 | 390 | 314 | 197 | 11 | 2 | 2 |
| Ratio to 2009 | 1.00 | 0.85 | 0.68 | 0.43 | --- | --- | --- |
| Market Share (%) | 2.97 | 2.30 | 1.98 | 1.44 | 0.10 | 0.02 | 0.02 |
| Strontium ranelate | | | | | | | |
| Months treat (n) | 1,082 | 1,207 | 1,369 | 1,225 | 896 | 234 | 13 |
| Ratio to 2009 | 1.00 | 1.12 | 1.27 | 1.13 | 0.83 | 0.22 | --- |
| Market Share (%) | 6.99 | 7.12 | 8.64 | 8.93 | 7.94 | 2.49 | 0.16 |
| Denosumab | | | | | | | |
| Months treat (n) | --- | --- | 17 | 340 | 670 | 1,194 | 1,615 |
| Ratio to 2012 | --- | --- | --- | 1.00 | 1.97 | 3.51 | 7.45 |
| Market Share (%) | --- | --- | --- | 2.48 | 5.94 | 12.73 | 19.28 |
| Parathyroid hormone | | | | | | | |
| Months treat (n) | 279 | 249 | 235 | 127 | 110 | 184 | 192 |
| Ratio to 2009 | 1.00 | 0.89 | 0.84 | 0.46 | 0.39 | 0.66 | 0.69 |
| Market Share (%) | 1.80 | 1.47 | 1.48 | 0.93 | 0.98 | 1.96 | 2.29 |
|  | | | | | | | |

In 2009 and from a total annual volume of 15,487 months of osteoporosis treatment dispensed, bisphosphonates alone accounted for 61.9% of the market share, and up to 76.9% when bisphosphonates in combination were added. Raloxifene accounted for 11.4% and ranelate for 7.0%, with minimal consumption of calcitonin (3.0%) and parathyroid hormones (1.8%). Single bisphosphonates experienced a fall of 2.7 fold (from 10,685 to 4,030 packages filled), while combinations fell by 2 fold. Use of raloxifene and parathyroid hormone was halved while calcitonin and strontium ranelate disappeared after the warnings and restrictions of use from the AEMPS. In 2015, and over a total volume of 8,375 months of treatment (roughly half of 2009), bisphosphonates -alone or in combination- still accounted for 66.6% of the market share, followed by denosumab (19.3%), which experienced a notable growth in the period. Raloxifene (12.1%) and parathyroid hormone (2.3%) maintained their market share although on a much smaller market than in 2009.

| Figure S11. Annual consumption of osteoporosis drugs 2009-2015 |
| --- |
|  |
|  |
| Figure S12. Market Share of osteoporosis drugs 2009-2015. |
|  |
